# COVID-19 global pandemic planning: Dry heat incubation and ambient temperature fail to consistently inactivate SARS-CoV-2 on N95 respirators

**DOI:** 10.1177/1535370220977819

**Published:** 2020-12-20

**Authors:** Douglas J Perkins, Robert A Nofchissey, Chunyan Ye, Nathan Donart, Alison Kell, Ivy Foo-Hurwitz, Timothy Muller, Steven B Bradfute

**Affiliations:** 1 Center for Global Health, Department of Internal Medicine, University of New Mexico Health Sciences Center, Albuquerque, NM 87131, USA; 2 Office of Research, University of New Mexico Health Sciences Center, Albuquerque, NM 87131, USA; 3 Department of Molecular Genetics and Microbiology, University of New Mexico Health Sciences Center, Albuquerque, NM 87131, USA

**Keywords:** Coronavirus, SARS-CoV-2, N95, masks, inactivation, decontamination

## Abstract

The ongoing pandemic of the novel severe acute respiratory syndrome coronavirus 2 (SARS-CoV-2) has placed a substantial strain on the supply of personal protective equipment, particularly the availability of N95 respirators for frontline healthcare personnel. These shortages have led to the creation of protocols to disinfect and reuse potentially contaminated personal protective equipment. A simple and inexpensive decontamination procedure that does not rely on the use of consumable supplies is dry heat incubation. Although reprocessing with this method has been shown to maintain the integrity of N95 respirators after multiple decontamination procedures, information on the ability of dry heat incubation to inactivate SARS-CoV-2 is largely unreported. Here, we show that dry heat incubation does not consistently inactivate SARS-CoV-2-contaminated N95 respirators, and that variation in experimental conditions can dramatically affect viability of the virus. Furthermore, we show that SARS-CoV-2 can survive on N95 respirators that remain at room temperature for at least five days. Collectively, our findings demonstrate that dry heat incubation procedures and ambient temperature for five days are not viable methods for inactivating SARS-CoV-2 on N95 respirators for potential reuse. We recommend that decontamination procedures being considered for the reuse of N95 respirators be validated at each individual site and that validation of the process must be thoroughly conducted using a defined protocol.

## Impact statement

Due to the ongoing coronavirus pandemic, there is a shortage of vital personal protective equipment such as N95 respirators. Simple decontamination methods, such as dry heat, are being considered to allow re-use of these masks. We demonstrate here that dry heat is not an effective method for disinfection of N95s inoculated with live SARS-CoV-2, the coronavirus that causes COVID-19. Furthermore, we find inconsistent inactivation of SARS-CoV-2-contaminated N95 masks after incubation at room temperature for 5 days. Therefore, additional methods for simple, inexpensive re-use of N95s are required.

## Introduction

The rapid global spread of the novel severe acute respiratory syndrome virus 2 (SARS-CoV-2), which causes coronavirus disease 2019 (COVID-19), has placed substantial strain on the supply and availability of personal protective equipment (PPE) for frontline healthcare personnel (HCP). Since caring for individuals with COVID-19 requires close contact, there is an enhanced risk of SARS-CoV-2 transmission in these settings. Although data on the rate of infections in HCP are not robust for all states, for those with more complete reporting, the Centers for Disease Control and Prevention (CDC) COVID-19 Response Team found that frontline healthcare workers are at an increased risk of infection with disproportionately higher numbers in women and minority ethnic groups.^1^ Data from the USA and UK among 2,025,395 community individuals and 99,795 frontline healthcare workers indicate that the risk of reporting a positive test for COVID-19 is at least three-fold higher in HCP and at least five-fold higher in Black, Asian, and minority ethnic healthcare workers. Importantly, inadequate PPE or reuse of PPE was associated with increased risk of COVID-19.^2^

Based on the global shortage of PPE due to surging demand and supply chain disruptions, particularly for N95 filtering facepiece respirators (FFRs), the CDC has provided revised guidance for the extended use and reuse (without decontamination) of FFRs.^3^ With the world-wide dissemination of COVID-19, there has been robust discussion of methods to inactive SARS-CoV-2 and decontaminate FFRs for their safe reuse. A number of previous studies investigated the impact of common decontamination methods on FFR performance, including ultraviolet germicidal irradiation (UVGI), hydrogen peroxide vapor (HPV), hydrogen peroxide gas plasma (HPGP), ethylene oxide (EtO), liquid hydrogen peroxide (LHP), microwave oven irradiation (MOI), microwave oven generated steam (MGS), moist heat incubation (MHI, pasteurization), and sodium hypochlorite (bleach, 0.6%).^4^^–^^7^ In late-March, 2020, we selected HPV as a viable reprocessing method, based on literature available at the time and subject matter expertise, began reprocessing FFRs at the University of New Mexico.^8^ Subsequently, the CDC put out crisis standards of care decontamination recommendations for the potential reuse of FFRs and suggested that HPV, UVGI, and moist heat are the most favorable methods.^9^ While each of these methods hold promise, the availability of specialized equipment to perform the decontamination process is not available in all environments, particularly those in resource-limited settings. As such, we investigated the ability of dry heat incubation (DHI) to inactivate SARS-CoV-2 on FFRs with different temperatures, times, and conditions since the method is: (1) widely available on a global scale, (2) inexpensive, (3) does not require consumable reagents to operate, (4) has the potential to efficiently reprocess considerable numbers of N95 respirators in a short period of time, and (5) does not appear to comprise the filtration efficiency of FFRs after multiple reprocessing cycles.^4,10^

An additional universally adaptable inactivation method that could allow reuse of potentially contaminated N95 respirators is allowing the FFRs to remain at ambient (room temperature) for a protracted period of time (days). Therefore, we also determined if keeping FFRs at ambient temperature for five days would inactivate SARS-CoV-2 on N95 respirators. Findings presented here have important implications about the potential risk associated with both DHI and ambient temperature as viable means of inactivating SARS-CoV-2 on N95 respirators.

## Materials and methods

### SARS-CoV-2 virus

SARS-related coronavirus 2 (SARS-CoV-2), isolate USA-WA1/2020, was deposited by the CDC and obtained through BEI Resources, NIAID, NIH (catalog number NR-52281). Virus was expanded and titered in Vero E6 cells (ATCC) by plaque assay. All experiments were conducted in a Biosafety Level 3 laboratory in the University of New Mexico Health Science Center with approved protocols.

### Inactivation experiments

3 M™ 1860S N-95 respirators were cut into pieces/coupons (1 cm × 1 cm) and placed in a sterile Petri dish. Coupons were then treated with UV light for 30 min on both sides (60 min total) in a biosafety cabinet, at a 10 cm distance from the UV light source, to inactivate any potential contaminating pathogens prior to the experimental procedures. The coupons were then sterilely transferred into 24 well tissue culture plates. The inside (absorbent) layer was inoculated with either high (1 × 10^5^ pfu) or low (3 × 10^3^ pfu) doses of SARS-CoV-2 and allowed to dry for 30 min. N95 coupons were placed on parchment paper or in the wells of a lid-covered 24-well tissue culture plate in a laboratory dry oven (Fisher Scientific™ Isotemp 500 series, model 5160) or at room temperature (22–23°C) for five days. N95 coupons were incubated at the indicated temperatures and times. In addition, DHI inactivation of high and low doses of SARS-CoV-2 was investigated using intact 3 M™ 8200 N95 respirators. Areas of the respirator were marked (1 cm × 1 cm) and inoculated with virus as above. The respirator was suspended by the elastic strips in the heat chamber at the various temperatures for 60 min. The inoculated areas were then cut out and placed into the wells of a 12-well tissue culture plate. To each well containing the N95 pieces, 1 mL of DMEM/10% FCS/1% Pen/Strep was added for 30 min to elute the virus. Eluant was removed with a 1 mL syringe and passed through a 0.22 µM filter onto Vero E6 cells growing in 1 mL of DMEM/10% FCS/1% penicillin/streptomycin.

### Assessment of viral inactivation

Vero E6 cells were incubated at 37°C for six days and cells were analyzed by microscopy for cytopathic effect (CPE). As negative controls, N95 coupons were not inoculated with SARS-CoV-2 and incubated at room temperature for the indicated times. As an additional negative control, N95 coupons were not inoculated with virus and subjected to DHI to ensure that any detected CPE was not due to respirator components. As positive controls, virus was added to N95 coupons and not subjected to DHI or ambient temperature incubation.

## Results

### Dry heat incubation of SARS-CoV-2 on N95 respirator coupons placed on parchment paper

**Figure 1.**
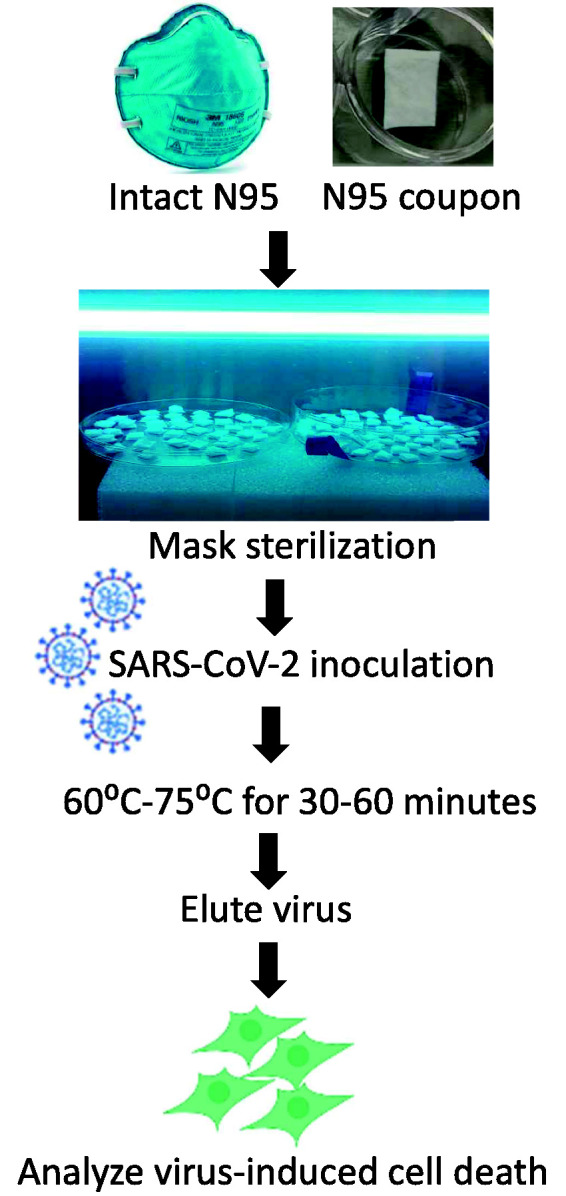
Experimental design. N95 coupons or whole (intact) respirators were UV-inactivated to ensure sterility and then inoculated with 1 × 10^5^ pfu or 1 × 10^3^ pfu of SARS-CoV-2. Samples were then placed in a dry heat chamber for various times at different temperatures. Virus was eluted from the mask material and incubated on Vero E6 cells for six days, at which time cells were analyzed for cytopathic effect (CPE). (A color version of this figure is available in the online journal.)

To assess whether DHI could inactivate SARS-CoV-2 on N95 respirators, 3 M™ 1860S N95 coupons (pieces cut into 1 cm × 1 cm containing all layers) were inoculated on the inner (absorbent) layer with SARS-CoV-2, placed on parchment paper, heated in a dry oven, and analyzed for viral inactivation (Figure 1). DHI at 60°C, 65°C, or 70°C for 30 min did not inactivate a high-dose inoculum (1 × 10^5^ pfu) of SARS-CoV-2 (Table 1). To ascertain whether increased heat and/or time could inactivate SARS-CoV-2 on the coupons and to validate the initial results, the experiment was repeated at 60°C, 65°C, 70°C, or 75°C for 30 or 60 min using both a high (1 × 10^5^ pfu) or low (3 × 10^3^ pfu) dose of SARS-CoV-2, respectively. None of the treatments conducted with the N95 coupons on parchment paper inactivated either a high or low dose inoculum of virus (Table 1).

**Table 1.** Dry heat incubation of high (1 × 10^5^ pfu) or low (3 × 10^3^ pfu) doses of SARS-CoV-2 on N95 coupons (3 M™ 1860S) placed on parchment paper.

Amount of virus	Temperature	Duration of incubation	Sample placement	# samples positive for CPE/total # samples
1 × 10^5^ pfu	60°C	30 min	Parchment paper	3/3* (3/3)
1 × 10^5^ pfu	65°C	30 min	Parchment paper	3/3* (3/3)
1 × 10^5^ pfu	70°C	30 min	Parchment paper	2/3* (3/3)
1 × 10^5^ pfu	75°C	30 min	Parchment paper	3/3
				
1 × 10^5^ pfu	70°C	60 min	Parchment paper	3/3
1 × 10^5^ pfu	75°C	60 min	Parchment paper	3/3
				
3 × 10^3^ pfu	60°C	30 min	Parchment paper	3/3
3 × 10^3^ pfu	65°C	30 min	Parchment paper	3/3
3 × 10^3^ pfu	70°C	30 min	Parchment paper	3/3
3 × 10^3^ pfu	75°C	30 min	Parchment paper	3/3
				
3 × 10^3^ pfu	70°C	60 min	Parchment paper	3/3
3 × 10^3^ pfu	75°C	60 min	Parchment paper	3/3
				
**Controls**				
1 × 10^5^ pfu	RT	30 min	Parchment paper	3/3* (3/3)
1 × 10^5^ pfu	RT	60 min	Parchment paper	3/3
				
3 × 10^3^ pfu	RT	30 min	Parchment paper	3/3* (3/3)
3 × 10^3^ pfu	RT	60 min	Parchment paper	3/3
				
No virus	RT	30 min	Parchment paper	0/3* (0/3)
No virus	RT	60 min	Parchment paper	0/3

*Indicates results from the first set of experiments.

### Dry heat incubation of SARS-CoV-2 on N95 coupons placed in tissue culture plate wells

To explore additional conditions, virus-inoculated N95 coupons were placed into the wells of a 24-well tissue culture plate and exposed to DHI at 70°C or 75°C for 30 or 60 min. Exposure of the coupons to 70°C or 75°C for 30 or 60 min, inactivated SARS-CoV-2 at both high and low doses of the virus (Table 2), suggesting that the surface on which the coupons are placed is an important experimental condition for successfully inactivating SARS-CoV-2.

**Table 2.** Dry heat incubation of high (1 × 10^5^ pfu) or low (3 × 10^3^ pfu) doses of SARS-CoV-2 on N95 coupons (3 M™ 1860S) placed in tissue culture plate wells.

Amount of virus	Temperature	Duration of incubation	Sample placement	# samples positive for CPE/total # samples
1 × 10^5^ pfu	70°C	30 min	Tissue culture plate	0/3
1 × 10^5^ pfu	75°C	30 min	Tissue culture plate	0/3
				
1 × 10^5^ pfu	70°C	60 min	Tissue culture plate	0/3
1 × 10^5^ pfu	75°C	60 min	Tissue culture plate	0/3
				
3 × 10^3^ pfu	70°C	30 min	Tissue culture plate	0/3
3 × 10^3^ pfu	75°C	30 min	Tissue culture plate	0/3
				
3 × 10^3^ pfu	70°C	60 min	Tissue culture plate	0/3
3 × 10^3^ pfu	75°C	60 min	Tissue culture plate	0/3
				
**Controls**				
1 × 10^5^ pfu	RT	30 min	Tissue culture plate	3/3
1 × 10^5^ pfu	RT	60 min	Tissue culture plate	3/3
				
3 × 10^3^ pfu	RT	30 min	Tissue culture plate	3/3
3 × 10^3^ pfu	RT	60 min	Tissue culture plate	3/3
				
No virus	RT	30 min	Tissue culture plate	0/3
No virus	RT	60 min	Tissue culture plate	0/3
				
No virus	70°C	30 min	Tissue culture plate	0/3
No virus	70°C	60 min	Tissue culture plate	0/3
				
No virus	75°C	30 min	Tissue culture plate	0/3
No virus	75°C	60 min	Tissue culture plate	0/3

### Dry heat incubation of SARS-CoV-2 on N95 coupons placed on parchment paper or tissue culture plate wells within the same heat chamber

Based on varying results using different materials on which the coupons were placed, we performed additional experiments in parallel within the same heat chamber. Coupons were placed on either parchment paper or within tissue culture plate wells and subjected to DHI at 70°C or 75°C for 60 min. As shown in Table 3, placement of the coupons on parchment paper failed to inactivate both high and low doses of the virus, as in the previous experiments (see Table 1). Conversely, placement of the coupons in tissue culture plate wells completely inactivated both high and low doses of SARS-CoV-2 at both 70°C and 75°C (Table 3), confirming that the surface material on which the coupons are placed has an important impact on the ability of DHI to inactivate SARS-CoV-2.

**Table 3.** Dry heat incubation of (1 × 10^5^ pfu) or low (3 × 10^3^ pfu) doses of SARS-CoV-2 on N95 coupons (3 M™ 1860S) placed on either parchment paper or tissue culture plate wells.

Amount of virus	Temperature	Duration of incubation	Sample placement	# samples positive for CPE/total # samples
1 × 10^5^ pfu	70°C	60 min	Parchment paper	3/3
1 × 10^5^ pfu	75°C	60 min	Parchment paper	3/3
				
3 × 10^3^ pfu	70°C	60 min	Parchment paper	3/3
3 × 10^3^ pfu	75°C	60 min	Parchment paper	3/3
				
1 × 10^5^ pfu	70°C	60 min	Tissue culture plate	0/3
1 × 10^5^ pfu	75°C	60 min	Tissue culture plate	0/3
				
3 × 10^3^ pfu	70°C	60 min	Tissue culture plate	0/3
3 × 10^3^ pfu	75°C	60 min	Tissue culture plate	0/3
				
**Controls**				
1 × 10^5^ pfu	RT	60 min	Parchment paper	3/3
1 × 10^5^ pfu	RT	60 min	Tissue culture plate	3/3
				
3 × 10^3^ pfu	RT	60 min	Parchment paper	3/3
3 × 10^3^ pfu	RT	60 min	Tissue culture plate	3/3
				
No virus	RT	60 min	Parchment paper	0/3
No virus	RT	60 min	Tissue culture plate	0/3

Note: Placement of the N95 coupons on parchment paper or in tissue culture plate wells was run concomitantly in the same heat chamber.

### Dry heat incubation of SARS-CoV-2 on intact N95 respirators

**Figure 2.**
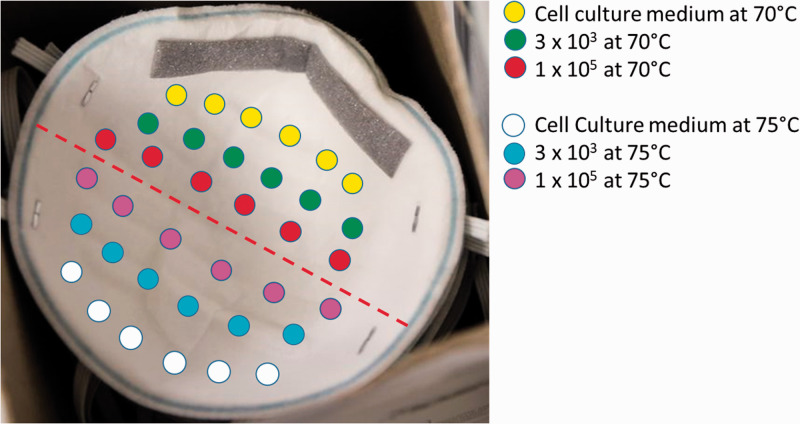
Virus placement on N95 mask. An N95 respirator was inoculated with SARS-CoV-2 and hung from the elastic straps in dry heat chamber for 60 min. The inoculated areas (1 cm × 1 cm) were then cut out and placed into a 24-well tissue culture plate for 30 min, at room temperature. Eluent was added to a 12-well plate with <80% confluent Vero E6 using a syringe and 0.22 µm sterile filter. The CPE was measured on day 6. (A color version of this figure is available in the online journal.)

To further explore different conditions associated with DHI inactivation of SARS-CoV-2 and to mimic an actual decontamination procedure, intact 3 M™ 8200 N95 respirators were inoculated with high (1 × 10^5^ pfu) or low (3 × 10^3^ pfu) doses of SARS-CoV-2 (or no virus, control) on the inner (absorbent) layer (Figure 2). The N95s were then suspended within the heat chamber by their elastic straps and subjected to DHI at 70°C or 75°C for 60 min. DHI failed to inactivate a high inoculum dose of SARS-CoV-2 at 70°C and 75°C, and at a low dose of the inoculum at 70°C (Table 4). Results for the low dose of SARS-CoV-2 inoculum at 75°C were variable with 4/6 of the inoculated areas containing infectious virus (Table 4). Collectively, these results indicate that DHI at 70°C or 75°C for 60 min is not a viable method for inactivating SARS-CoV-2 on the intact N95 respirator model tested.

**Table 4.** Dry heat incubation of high (1 × 10^5^ pfu) or low (3 × 10^3^ pfu) doses of SARS-CoV-2 on intact 3 M™ 1860 N95 respirators.

Amount of virus	Temperature	Duration of incubation	Sample placement	# samples positive for CPE/total # samples
1 × 10^5^ pfu	70°C	60 min	Hanging	6/6
1 × 10^5^ pfu	75°C	60 min	Hanging	6/6
				
3 × 10^3^ pfu	70°C	60 min	Hanging	6/6
3 × 10^3^ pfu	75°C	60 min	Hanging	4/6
				
**Controls**				
1 × 10^5^ pfu	RT	60 min	Hanging	3/3
				
3 × 10^3^ pfu	RT	60 min	Hanging	3/3
				
No virus	RT	60 min	Hanging	0/6
No virus	70°C	60 min	Hanging	0/6
No virus	75°C	60 min	Hanging	0/6

**Figure 3.**
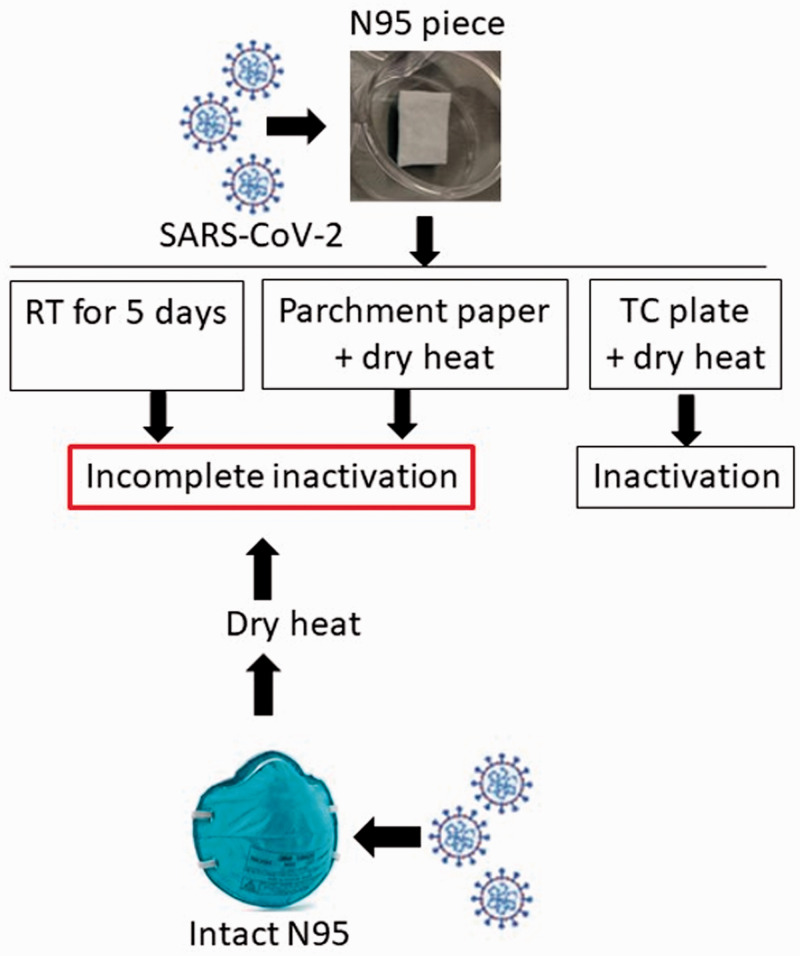
Summary. Neither dry heat incubation for 30–60 min nor room temperature incubation for five days consistently inactivated SARS-CoV-2 on N95 coupons or intact masks. (A color version of this figure is available in the online journal.)

### Inactivation on SARS-CoV-2 at ambient (room) temperature

A highly practical and low-cost method for decontamination of N95 respirators is to simply store them at room temperature until the virus is degraded. To test the feasibility of this method, 3 M™ 1860S FFR coupons were inoculated with 1 × 10^5^ pfu of SARS-CoV-2 (high dose) and incubated in 24-well tissue culture plate wells for five days at room temperature, followed by virus isolation and incubation on Vero E6 cells to detect live virus. Two independent experiments were performed. In the first experiment, SARS-CoV-2 was completely inactivated (3/3 coupons) after five days at room temperature. To confirm these results, we performed a second set of experiments which revealed that 5/9 coupons contained live virus at five days under identical conditions (Table 5). Therefore, allowing N95s to remain at ambient temperature for five days does not appear to be a reproducibly viable method for reprocessing since there was inconsistent inactivation of a high dose of SARS-CoV-2.

**Table 5.** Survival of a high dose of SARS-CoV-2 (1 × 10^5^ pfu) on N95 coupons (3 M™ 1860S) placed in tissue culture plate wells at room temperature for five days.

Amount of virus	Temperature	Duration of treatment	Sample placement	# samples positive for CPE/total # samples
1 × 10^5^ pfu	RT	5 days	Tissue culture plate	0/3* (5/9)
				
**Controls**				
1 × 10^5^ pfu	4°C	5 days	Eppendorf tube	0/3* (9/9)

*Indicates results from the first set of experiments.

## Discussion

The ability to reprocess FFRs with inexpensive and widely available methods for safe reuse is an important strategy to address challenges when facing supply shortages. Such methods are particularly vital for low-resource environments in which specialized equipment and reagents may not be available. Since many settings around the globe have access to heat chambers for standard hospital and laboratory use, we began exploring dry heat as a potential reprocessing option in early-April 2020 as a low-cost and readily available method to reprocess N95 respirators. At that time, there were data showing that dry heat at 75°C for 30 min did not significantly impact the filtration efficiency and pressure drop after multiple reprocessing cycles, and that these conditions inactivated *Escherichia coli* bacteria (>99%) on these respirators.^10^ Other studies have described inactivation of surrogate viruses with DHI.^7,11^ Our findings suggest that the use of surrogate pathogens may not faithfully represent the efficacy of various methods on SARS-Cov-2 inactivation and that experimental variations could affect inactivation efficacy. Although inactivation of SARS-CoV-2 was not determined in that study, several other previous studies performed on the related SARS-CoV or Middle Eastern Respiratory Syndrome coronavirus (MERS) indicated that thermal disinfection of a liquid solution at 60°C for 30 min and 65°C for 10 to 15 min reduced viral infectivity by ≥ 4 log_10_.^12–14^

We reasoned that viral inactivation in suspension may not necessarily reflect inactivation of the virus on the solid surface of N95 respirators. As such, we initially explored DHI at several different temperatures and time periods, and on different substrates (i.e. parchment paper and tissue culture plate wells). In our initial series of experiments, we used N95 coupons, instead of intact masks, due to national supply chain shortages. For these investigations, we placed SARS-CoV-2 on the inner layer of the mask pieces since the virus is not readily absorbed to the outer (hydrophobic) layer. Thus, this experimental paradigm is representative of contamination of the inner, clean side of the mask against the user’s face. Results presented here show that DHI failed to inactivate both high (1 × 10^5^ pfu) and low (3 × 10^3^ pfu) doses of SARS-CoV-2 at a range of temperatures (60°C to 75°C) for up to 60 min when the coupons were placed on parchment paper. Conversely, DHI consistently inactivated high and low doses of the virus at 70°C and 75°C for both 30 and 60 min when the N95 material was placed into the wells of tissue culture plates. These results suggest that transfer of heat through the material on which the N95 coupons were placed appears to have a significant impact on the ability of dry heat to inactivate the virus. Our findings further indicate that conditions for heat inactivation of the virus in liquid suspension appear to differ from those required for inactivation of SARS-CoV-2 when fixed on N95 material. The results obtained when placing the coupons in tissue culture plate wells are similar to a recent publication showing that dry heat at 70°C for 30 min reduced SARS-CoV-2 virus titers (TCID_50_/mL of media) from 10^5^ to ∼10^2^ on N95 fabric, and at the same temperature for 60 min, reduced the virus to an undetectable level;^15^ a similar study found inactivation of >10^4.8^ TCID_50_ SARS-CoV-2 at various levels of humidity at 70°C for 60 min.

Once we completed the series of experiments on N95 coupons, we then explored DHI as a means of inactivating SARS-CoV-2 spotted onto intact respirators since this process mimics an actual decontamination procedure for potential re-use. For these experiments, we suspended 3 M™ 8200 N95s in the heat chamber at either 70°C or 75°C for 60 min. DHI failed to inactivate a high dose of SAR-CoV-2 (1 × 10^5^ pfu) at either 70°C or 75°C for 60 min. Treatment of respirators with dry heat for 60 min at 70°C also failed to inactivate a low dose of the virus (3 × 10^3^ pfu), whereas DHI at 75°C failed to inactivate four out of six regions at that dose. These results suggest that DHI, under the conditions examined and on the model of respirator tested, is not a reliable and robust method of inactivating SARS-CoV-2 on intact respirators (Figure 3). Others have suggested the use of moist heat (similar heat ranges conducted here but in the presence of high humidity) as a method to inactivate viruses on masks while preserving mask integrity.^16^ Studies have found that changes in relative humidity affect efficacy of heat inactivation for other viruses.^17^ We did not test this approach as it requires ovens that can regulate humidity, and these devices are not as widespread as dry ovens. However, the use of moist heat for N95 inactivation and reuse is another possible approach that should be tested using live SARS-CoV-2 on intact masks.

An ideal means of decontaminating N95 respirators would be allowing the FFRs to sit at room temperature for a protracted period. Several studies have examined the survival of SARS-CoV-2 on a number of surfaces at room temperature and found that survival can vary from hours to days, depending on the type of surface tested.^18,19^ As such, we explored inactivation of a high dose of SARS-CoV-2 by placing N95 coupons in tissue culture plate wells at room temperature for five days. Our results show that SARS-CoV-2 was not consistently inactivated under these conditions, as illustrated by 5 out of 12 coupons showing positivity for the virus at five days. These data indicate that SARS-CoV-2 can survive on N95 mask material for at least five days, although it appears that some viral inactivation does occur (Figure 3). It is reasonable to infer that the virus will also survive on intact FFRs, and that allowing the masks to sit at room temperature for five days does not provide a consistently safe means of SARS-CoV-2 inactivation for the end-user. However, given that some mask coupons were inactivated at this time point, it is possible that a longer incubation period (for example, 7–10 days) could result in complete inactivation. Additional experiments are required to confirm this hypothesis and should be pursued, as this would be a simple, inexpensive, and universally available method for N95 decontamination and reuse.

One potential limitation is that the experiments were conducted by placing SARS-CoV-2 on the interior surface of the N95 coupons and intact respirators: this approach was taken because the virus solution beaded up on the exterior surface of the mask and was not practical for the experimental procedures. It is possible that the results could differ if the virus was placed on the outside (hydrophilic layer) of the respirator. However, our approach is particularly relevant to decontamination and re-use procedures in which the wearer of the mask was infected with SARS-CoV-2 (either symptomatic or asymptomatic) and in situations in which the respirator interior becomes inadvertently contaminated during donning or doffing. Furthermore, this information is relevant to healthcare workers sharing DHI-treated N95 masks, where incomplete viral inactivation could result in the transmission of SARS-CoV-2 between individuals. It is also possible that use of moist heat could be effective and is an avenue that should be pursued. We did not analyze virus in the presence of saliva or nasal secretions, which would be more physiologic.

Collectively, our data demonstrate that subtle intricacies in the experimental protocols (i.e. parchment paper vs. tissue culture plate wells) have a substantial impact on the survival of authentic SARS-CoV-2 on N95 coupons. It is therefore critical to conduct and validate experiments for potential decontamination methods on intact FFRs for identical models and in the exact manner that will be applied in the real-world decontamination of the PPE. Our data demonstrate that dry heat incubation is not consistently effective at eliminating SARS-CoV-2 on contaminated N95 respirators. Furthermore, we show that SARS-CoV-2 can survive for up to five days at room temperature on N95 coupons. Based on findings presented here, inactivation of SARS-CoV-2 by DHI or ambient temperature for five days are not viable decontamination options for safe re-use.
